# What Is a Genome?

**DOI:** 10.1371/journal.pgen.1006181

**Published:** 2016-07-21

**Authors:** Aaron David Goldman, Laura F. Landweber

**Affiliations:** 1 Department of Biology, Oberlin College, Oberlin, Ohio, United States of America; 2 Department of Ecology and Evolutionary Biology, Princeton University, Princeton, New Jersey, United States of America; 3 Departments of Biochemistry & Molecular Biophysics and Biological Sciences, Columbia University, New York, New York, United States of America; Dalhousie University, CANADA

## Abstract

The genome is often described as the information repository of an organism. Whether millions or billions of letters of DNA, its transmission across generations confers the principal medium for inheritance of organismal traits. Several emerging areas of research demonstrate that this definition is an oversimplification. Here, we explore ways in which a deeper understanding of genomic diversity and cell physiology is challenging the concepts of physical permanence attached to the genome as well as its role as the sole information source for an organism.

## Introduction

**Fig 1:**
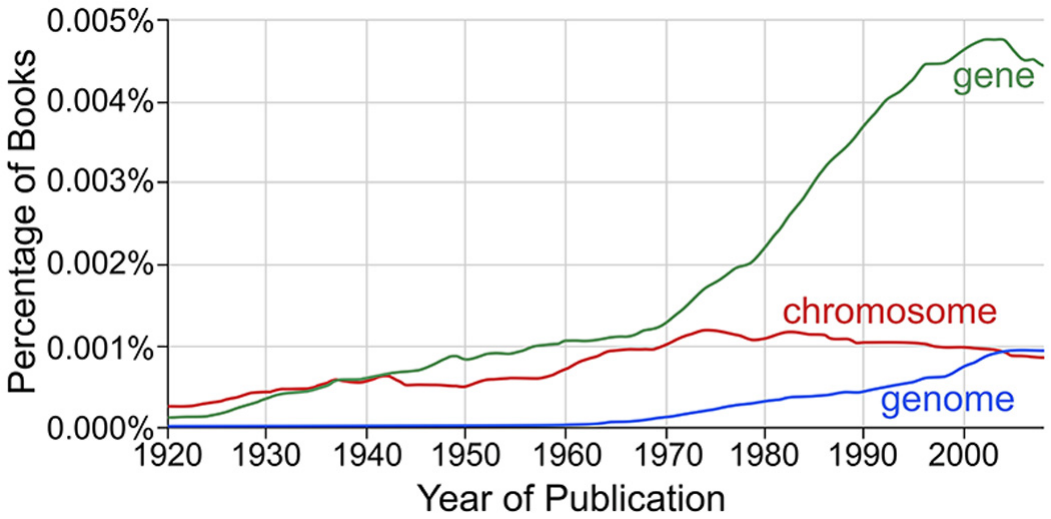
The change in usage of the term “genome” compared to related terms. A Google ngram [6] analysis shows the case-insensitive occurrences of the terms “gene,” “genome,” and “chromosome” in the corpus of books in English from 1920 to 2008. The data are smoothed by a three-year moving average. The term “genome” was coined in 1920 [1], and many sources, including the Oxford English Dictionary, attribute the word to a portmanteau of the words “gene” and “chromosome,” although this etymology is disputed [1]. The term took decades to enter popular usage and only achieved its current level of usage by the turn of this century.

The term genome was coined in 1920 to describe “the haploid chromosome set, which, together with the pertinent protoplasm, specifies the material foundations of the species” [1]. The term did not catch on immediately (Fig 1). Though Mendelian genetics was rediscovered in 1900, and chromosomes were identified as the carriers of genetic information in 1902 [2], it was not known in 1920 whether the genetic information was carried by the DNA or protein component of the chromosomes [3]. Furthermore, the mechanism by which the cell copies information into new cells [4] and converts that information into functions [5] was unknown for several decades after the term “genome” was coined.

Today, however, we are awash in genomic data. A recent release of the GenBank database [7], version 210.0 (released on October 15, 2015), contains over 621 billion base pairs from 2,557 eukaryal genomes, 432 archaeal genomes, and 7,474 bacterial genomes, as well as tens of thousands of viral genomes, organellar genomes, and plasmid sequences (http://www.ncbi.nlm.nih.gov/genome/browse/, on December 13, 2015). We also now have much broader and more detailed understandings of how the genome is expressed and how different biological and environmental factors contribute to that process. Even so, almost a century after coining the term, the standard definition of the genome remains very similar to its 1920 predecessor. For example, on its Genetics Home Reference website, the National Institutes of Health (NIH) definition reads: “An organism’s complete set of DNA, including all of the genes, makes up the genome. Each genome contains all of the information needed to build and maintain that organism” (http://ghr.nlm.nih.gov/handbook/hgp/genome, on February 1, 2016).

With a greater understanding of genomic content, diversity, and expression, we can now reassess our basic understanding of the genome and its role in the cell. For example, closer scrutiny of the NIH definition reveals that its two halves are mutually exclusive; that is, the “complete set of DNA” cannot be “all of the information needed to build and maintain (an) organism.” Of course, this was probably meant to be a simplified definition for both scientists and nonscientists. While it is useful to continue thinking of the genome as a physical entity encoding the information required to maintain and replicate an organism, our present understanding shows that this definition is incomplete.

## Examples of Physical Transience in Genomes

Many diverse genetic systems challenge the material definition of the genome as “the complete set of *chromosomes*” [1] or “an organism’s complete set of *DNA*” (http://ghr.nlm.nih.gov/handbook/hgp/genome). Perhaps the most familiar and straightforward example of a genome’s physical impermanence occurs in the retroviral infection cycle. Upon infection, retroviruses convert their single-stranded RNA genomes into double-stranded DNA. These intermediate DNA copies of the genome are integrated into the host cell and, thus, no longer constitute a separate physical entity from the host’s genome. As an integrated DNA sequence, transcription into mRNA can both express retroviral genes and also reconstitute the original single-stranded (ss)RNA genome. Other types of viruses share similar features. Many temperate phages and viruses integrate into the host’s genome, removing themselves and lysing the host cell only after certain conditions are met. The hepadnaviruses, including Hepatitis B, infect the cell as double-stranded DNA, but are transcribed after infection into single-stranded RNA and subsequently follow a similar course as the retroviruses, wherein they are reverse transcribed back into DNA [8].

The chemical conversions of these genomes between different nucleic acids offer cogent examples that challenge our assumption of the physical permanence of genomes. It is tempting to explain this physical transience as another eccentric quirk of viruses. Many viruses, after all, do not have genomes composed of double-stranded DNA, a feature that already flouts the NIH definition given earlier. But an equally cogent example of the physical impermanence of a genome is found in the eukaryotic genus *Oxytricha* [9–11], a group of ciliates that are distantly related to *Tetrahymena* and *Paramecium* [12].

**Fig 2:**
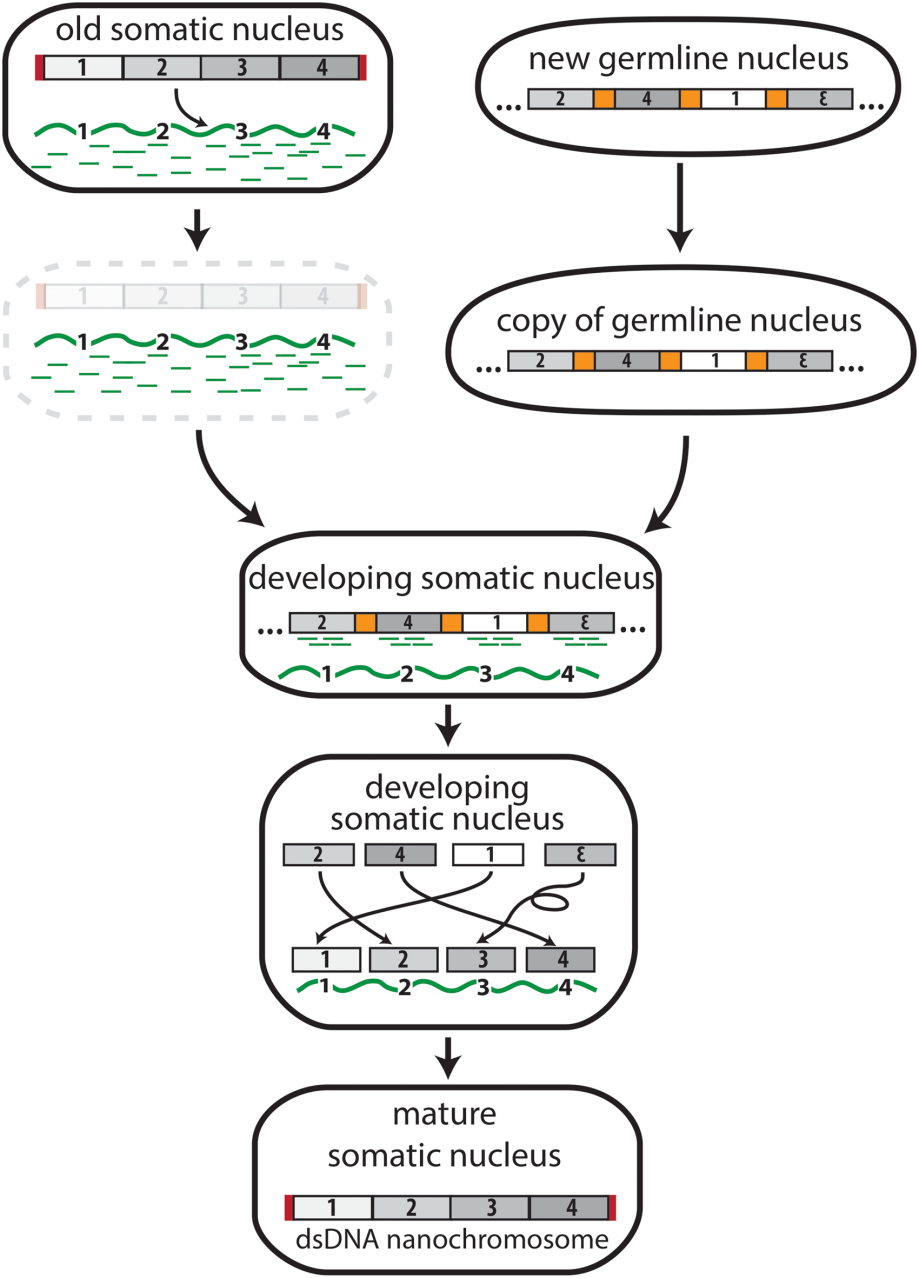
The transfer of genomic information from DNA to RNA in *Oxytricha trifallax*. The physical transition of genomic information from DNA to RNA and back to DNA occurs after mating in the ciliate, *Oxytricha trifallax*. RNA templates (wavy green line) and piRNAs (green dashes) derive from RNA transcripts of the previous generation’s somatic DNA nanochromosomes before the old somatic nucleus degrades. A mitotic copy of the new, zygotic germline genome provides precursor DNA segments (numbers 1–4) that are retained in the developing somatic nucleus through piRNA associations and rearranged according to the inherited RNA templates. This step sometimes reorders or inverts precursor segments to build the mature DNA molecule. The number of copies of each new nanochromosome is also influenced by the concentration of RNA templates supplied by the previous somatic genome during development. Red rectangles represent telomeres added to the ends of somatic chromosomes. Only one representative nanochromosome (of over 16,000 in *Oxytricha*) is shown for simplicity, and it derives from a representative locus containing 4 scrambled precursor segments in the germline genome.

Like other ciliates, *Oxytricha* possesses two distinct versions of its genome, a germline version and a somatic version. *Oxytricha*’s germline genome is an archive of approximately 1 Gb of DNA sequence containing approximately one-quarter million embedded gene segments. These DNA pieces assemble following sexual recombination to form the somatic, expressed chromosomes (Fig 2). Thousands of these gene segments are present within the germline chromosomes in a scrambled order or reverse orientation, such that their reassembly requires translocation and/or inversion with respect to one another [13]. The resulting somatic genome, containing protein-coding sequences in the correct order, contains just 5%–10% the original sequence of the germline genome. This somatic genome resides on over 16,000 unique “nanochromosomes” that typically bear single genes and have an average size of just 3.2 kb [14]. These nanochromosomes also exist in high copy number, averaging approximately 2,000 copies per unique chromosome [14,15].

Much of the information required to reproduce the somatic genome derives from RNA rather than DNA. Long, RNA-cached copies of somatic chromosomes from the previous generation provide templates to guide chromosome rearrangement [16]. Germline transposases participate in the whole process, probably by facilitating DNA cleavage events [17,18] that allow genomic regions to rearrange in the order according to the RNA templates [16]. Experimental introduction of long artificial RNAs can reprogram a developing *Oxytricha* cell to follow the order of gene segments specified by the artificial RNA templates, rather than the wild-type chromosome.

RNA performs other essential roles in building *Oxytricha*’s somatic genome. Millions of small, 27-nt piRNAs, which also derive from the previous generation’s somatic genome, mark and protect the retained DNA regions in the new zygotic germline that assemble (according to the RNA template) to form the new somatic genome [19,20]. In addition, the relative abundance of the long template RNAs also establishes chromosome copy number in the daughter cells [17]. Because these RNA templates derive from the previous generation’s somatic genome, this means that both the genomic sequence and chromosome ploidy are inherited from the old somatic nucleus to the new somatic nucleus through information transfer from DNA to RNA and back again to DNA.

These examples of physical transience in genomes show that a genome’s chemical composition and stability are not necessarily fixed requirements at all times in every organism. Synthetic biologists have further demonstrated this point through the chemical synthesis of viral [21,22] and bacterial [23] genomes. Prior to the chemical synthesis of these DNA chromosomes, the genomes existed in a purely informational state as nucleotide sequences in a computer file. In these cases, the genome of the virus or cell is not transferred from one type of nucleic acid to another, but from a physical DNA molecule to a non-physical nucleotide sequence and back again to a physical DNA molecule. Though this example is not a naturally occurring phenomenon, it provides a straightforward demonstration that the information content of the genome is more important than its physical permanence. Therefore, the concept of informational supremacy that is used to define genomes, e.g., “all of the information needed to build and maintain that organism,” also deserves further scrutiny.

## Extra-Genomic Information

Information is both an essential concept that underpins our understanding of a genome’s function and a notoriously difficult concept to define. The genome contains information, but so do other constituents of the cell. A typical and uncontroversial view is that the genome carries information but requires the presence of proteins, ribosomal RNAs, and transfer RNAs in the cell for the meaningful conversion of genomic information to molecular function. Indeed, the construction of synthetic genomes mentioned earlier required transplantation of the chemically synthesized genome into a pre-existing cell [23]. Evidence for heritable information beyond the genome has also been known since the 1960s [24]. A greater understanding of molecular biology has revealed that extra-genomic sources of information are not only required to read the genome but can influence the information encoded within the genome [25].

Epigenetic control of gene regulation provides a subtler—but in many ways more cogent—example of extra-genomic information. DNA methylation [26,27], histone modification encoding chromatin [28,29], and certain proteins (e.g., [30,31]) and noncoding RNAs [32,33], including *Oxytricha’s* noncoding RNAs described in the previous section [17,18,20], all offer platforms that permit information transfer across generations, while seeming to bypass the DNA genome. It has not yet been shown whether epigenetic information can persist over scales of evolutionary time, but it is clear that many if not most genomes have evolved a capacity for epigenetic control. This makes such genomes sensitive to external information that they do not encode, which, in turn, should influence their ability to adapt to changing environments while, in some cases, preserving the ability to revert to the former wild-type genome. This is epitomized by the genome duality in *Oxytricha*, in which millions of small and long noncoding RNAs sculpt and decrypt the information in its somatic epigenome, while the germline genome provides a more stable archive.

A second example of extra-genomic information has come by way of genome-wide association studies, which have identified correlations between many phenotypic traits and genetic variants [34]. In doing so, such studies have also revealed the so-called “missing heritability” problem, that genetic variation does not always account for 100% of the measured heritability, let alone the observed phenotypic variance, in many complex traits. In many cases, this missing heritability can be explained as a lack of statistical power due to low phenotypic impact of the genetic variation or low frequency in the population [35]. The missing heritability can also be explained, however, by a gene–environment interaction, such that the genes may only encode a trait that is expressed under certain environmental conditions [36,37]. In this example, genomes do not necessarily encode all of the information of the cell, but rather a set of potential states that may be realized through interaction with different environments.

As these examples demonstrate, the way in which the information content of the genome becomes realized as functions and phenotypes depends on other cellular constituents as well as the environment. The ability of genomes to be affected by this external information is, itself, encoded on the genome. In this way, genomes are not a sole source of cellular information, but rather a more expansive archive of possible states that can be generated through interactions with internal and external factors.

## Conclusion

Many biologists already know that the genome is not always best defined as “all of the information needed to build and maintain” a cell or an organism. While this definition is useful in the context of an online glossary for the public, it is, by necessity, an oversimplification. But if a genome is not a complete set of DNA containing all of the information needed to build and maintain the organism, then what is it?

We have demonstrated through examples from retroviruses, the microbial eukaryote *Oxytricha*, and synthetic biology that the genome can change its physical character while still maintaining the necessary information encoded within it. We also describe examples in which non-genomic factors can alter the way in which the information within the genome translates to molecular functions and phenotypes. These examples suggest a more expansive definition of the genome as an informational entity, often but not always manifest as DNA, encoding a broad set of functional possibilities that, together with other sources of information, produce and maintain the organism. Whether or not even this definition stands up to future discoveries remains to be seen.
